# Transient weakening of the blood-brain barrier in neonatal mice

**DOI:** 10.1186/s12987-026-00858-7

**Published:** 2026-08-11

**Authors:** Amira Sayed Hanafy, Dirk Dietrich

**Affiliations:** 1 Institute for Cellular Neurosciences II & Department of Neurosurgery, University Hospital Bonn, Bonn, Germany; 2 Department of Pharmaceutics, Institute of Pharmacy, University of Bonn, Bonn, Germany

**Keywords:** Blood-brain barrier, Tight junctions, Striatum, Brain development, Perivascular cells, Neonatal brain, Neurovascular unit

## Abstract

**Background:**

Tight junctions (TJs) are a major structural component of the blood-brain barrier (BBB), contributing to brain homeostasis by restricting paracellular diffusion. Although BBB maturation begins during embryogenesis, the timing and dynamics of functional barrier maturation perinatally remain unclear. This question is particularly relevant for the striatum, a metabolically demanding brain region that undergoes rapid postnatal maturation and is vulnerable to neonatal injury.

**Methods:**

We investigated developmental dynamics of BBB tracer permeability in mouse striatum using in situ microperfusion of capillaries (ISMICAP) combined with two-photon microscopy. Small-molecule tracers were applied from late embryonic (E18) to adolescent (P25) stages. Bovine serum albumin (BSA) was applied as a macromolecular tracer at P2 and P25.

**Results:**

Small-molecule tracers, namely 7-hydroxycoumarin-3-carboxylic acid (7HCC), sulforhodamine 101 (SR101), and biocytin-tetramethylrhodamine, revealed a pronounced but transient increase in extravascular fluorescence during the neonatal phase (P0-P2), followed by progressive restriction by P12 and P25. During the neonatal permeability window, 7HCC labeled perivascular cells located ~ 2.8 μm from the endothelium and distinct from NT500/525-labeled pericytes, whereas SR101 accumulated within endothelial cytoplasm, indicating tracer-specific vascular and perivascular accumulation patterns. The membrane probe FM1-43 showed a similar temporal pattern, with enhanced diffusion to abluminal membranes and labeling of pericyte-like mural cells. In contrast, BSA showed low extravascular fluorescence at P2 comparable to P25, indicating that increased neonatal small-molecule permeability did not extend to BSA-sized macromolecules. At P25, small-molecule tracer permeability was higher in the striatum than in the cortex, whereas FM1-43 showed the opposite regional pattern.

**Conclusions:**

BBB maturation in the striatum is not a linear tightening process but includes a transient neonatal phase of increased TJ-associated permeability to small-molecule tracers, while remaining restrictive to BSA-sized macromolecules. Although tracer-specific vascular and perivascular accumulation patterns were observed, the overall temporal profile supports a discrete perinatal window of decreased barrier restriction. This dynamic permeability window may reflect physiological remodeling of barrier function during the perinatal transition. Defining this window mechanistically may improve understanding of neonatal brain vulnerability and may inform strategies for temporally targeted CNS drug delivery.

## Introduction

The blood-brain barrier (BBB) is a dynamic interface that regulates the exchange of molecules between the circulation and brain parenchyma. It is formed by endothelial cells (ECs) interconnected by tight junctions (TJs) and adherens junctions, ensheathed by pericytes (PCs) and astrocytic endfeet, and supported by the basal lamina [1]. Together with neurons, these cells constitute the neurovascular unit (NVU), which integrates vascular and neural function [2]. This specialized structure enables selective transport through carriers and efflux pumps while restricting passive diffusion, thereby maintaining the ionic and metabolic milieu required for neuronal activity [2].

TJs are formed at the contact sites between adjacent ECs, where transmembrane adhesion proteins from opposing membranes interact in a tightly regulated manner [3]. Each TJ protein contributes both intramembranous domains embedded within the lipid bilayer, which restrict diffusion along the endothelial membrane from the luminal to the abluminal side of ECs [4, 5], and extramembranous domains that extend into the intercellular cleft to seal it and limit paracellular diffusion. Together, these junctional domains contribute to the restriction of paracellular and membrane-associated diffusion, while BBB function is further shaped by transcellular transport systems, vesicular trafficking, efflux pumps, carrier-mediated transport, and interactions within the NVU. The BBB emerges early during brain angiogenesis, when vascular sprouts invade the neural tube and differentiate into cerebral capillaries. At first, these vessels have large diameters, irregular morphology, diaphragmed fenestrae, and only rudimentary TJ contacts [6]. As development proceeds, ECs progressively acquire the hallmarks of a restrictive barrier, where fenestrae disappear, vessels become narrower and more regular in shape, and the strands of TJs elongate, interconnect, and increase in complexity until reaching the adult conformation [7, 8]. It was found that TJ-associated particle density increases substantially during late embryogenesis and the early postnatal stage, coinciding with a progressive restriction of paracellular permeability [7, 8]. Despite this structural evidence of tightening, the functional maturity of the fetal and neonatal BBB has long been debated. Early tracer studies suggested that plasma proteins enter the fetal brain in higher amounts than in adults [9, 10], whereas immunohistochemical analyses of endogenous plasma proteins reported little to no leakage, arguing for an already restrictive barrier [11]. These discrepancies likely reflect technical limitations, species differences in developmental timing, and historically limited access to standardized permeability readouts. Recent longitudinal in vivo two-photon imaging approaches using neonatal cranial windows have begun to provide physiological measurements with temporal and spatial resolution in intact cortex [12, 13].

Barrier tightness, however, cannot be inferred solely from protein expression. Functional tightness arises from the integrated contribution of structural junctions, transporter activity, and cellular interactions within the NVU. Although many studies suggest that restriction to macromolecules is established prenatally, permeability to small molecules appears more variable and may only decline perinatally [12, 13]. This view is tempered by a comprehensive review that cautions many early claims of developmental protein leakage may have been influenced by methodological limitations [14]. The conflicting reports and methodological limitations mean that the precise temporal dynamics of BBB maturation remain unresolved. Importantly, barrier tightening does not occur uniformly but progresses in a regionally heterogeneous manner during ongoing angiogenesis [15].

Despite the significant advances in molecular characterization, our functional understanding of BBB maturation remains incomplete. In particular, it remains unclear how functional BBB permeability to small molecules changes during early postnatal development, and to what extent these changes reflect junctional maturation, transcellular transport pathways, or tracer-specific handling by endothelial and perivascular cells. This question has strong biological and clinical relevance. The neonatal period represents a critical window of brain vulnerability: newborns are highly susceptible to systemic infections, hypoxic-ischemic injury, and inflammatory insults, all of which are exacerbated by BBB disruption [16]. Furthermore, neonates can be exposed to CNS therapeutics whose distribution is affected by BBB permeability and efflux properties that may differ from those in adults [17]. Thus, fluctuations in BBB permeability and tracer access perinatally could alter brain exposure to endogenous metabolites and exogenous agents, with long-term consequences for neurodevelopment.

The striatum provides a particularly relevant brain region for addressing these questions. It is among the most densely vascularized brain regions [18] and exhibits high metabolic demand during early development. It is also selectively vulnerable in neonatal hypoxic-ischemic encephalopathy [19], a leading cause of newborn brain injury, and is affected in inflammatory and infectious conditions early in life. Recent evidence shows that striatal output actively regulates the maturation of upstream cortical circuits, emphasizing its dynamic role in postnatal brain development [20]. Functionally, striatal circuits undergo rapid maturation in neonates, supporting motor and cognitive development [21]. In addition to this biological relevance, the striatum provides a reproducible anatomical region for standardized ISMICAP experiments across embryonic and postnatal stages, allowing longitudinal comparison of barrier function within the same region throughout development. These neurovascular and methodological characteristics make the striatum well suited for studying developmental BBB permeability dynamics.

Another unresolved question is whether BBB properties are homogeneous across brain regions. Anatomical and molecular studies reveal substantial interregional endothelial heterogeneity in gene expression, transporter profiles, and junctional composition [22, 23], as well as structural and functional differences within the NVU [24]. However, functional permeability comparisons across brain regions remain sparse. Investigating such heterogeneity is of great importance because regional differences in barrier properties could affect localized metabolite availability, signaling dynamics, and drug distribution, thereby influencing circuit maturation and vulnerability.

In vivo cranial window imaging provides an essential physiological perspective, but it is typically limited to accessible cortical regions and offers less experimental control over luminal composition and local delivery parameters [12, 13]. To address these questions, we applied in situ microperfusion of capillaries (ISMICAP), a technique we recently developed as a high-resolution functional assay of the BBB [5]. ISMICAP combines micro-impalement of venules in acute brain slices with two-photon microscopy, enabling controlled intravascular delivery of tracers and real-time monitoring of their cellular and subcellular interaction with ECs as well as perivascular structures. Acute slices, prepared from freshly isolated brains, remain viable for several hours under oxygenated conditions and preserve the native cytoarchitecture of the NVU [5]. This approach allows precise spatial control and direct visualization of intravascular and extravascular tracer access as well as vascular/perivascular tracer localization under controlled experimental conditions, without interference from systemic metabolism and clearance.

In this study, we systematically investigated the developmental dynamics of BBB tracer permeability in the striatum using ISMICAP. Using small-molecule probes with distinct physicochemical properties and vascular-perivascular labeling patterns, we quantified extravascular tracer diffusion, abluminal access and cellular uptake accumulation across embryonic (E18), neonatal (P0-P2), early postnatal (P12), and adolescent (P25) stages. Additionally, we included macromolecular bovine serum albumin (BSA) to address the long-standing question of whether protein-sized tracers show enhanced leakage during early development. To assess regional specificity, we further compared tracer permeability profiles in the striatum and cortex at P25. Our results identify an abrupt, yet transient, increase in small-molecule BBB tracer permeability and vascular/perivascular tracer accumulation in neonates, followed by rapid normalization, thus defining a discrete developmental window of altered barrier function. Moreover, we uncover regional differences in tracer permeability profiles, highlighting the importance of considering brain region when analyzing BBB dynamics. Together, these findings establish a functional framework for understanding developmental BBB maturation and provide a basis for exploring how transiently altered barrier properties may contribute to neonatal brain vulnerability.

## Methods

### Animals

C57BL/6NCrl mice (Charles River Laboratories) were used in all experiments across four age groups: embryos (E18), neonates (P0-P2), early postnatal (P12), and adolescent (P25). Embryonic, neonatal, and early postnatal groups included both sexes, while only male mice were used at the adolescent stage (P25) to maintain consistency with previously acquired datasets generated under identical experimental conditions. E18 was selected as a defined late prenatal stage because the timing of birth varied between litters, with delivery occurring between E19 and E21. Embryonic and neonatal mice were obtained from pregnant dams via timed breeding by the supplier. All experiments were performed in accordance with the guidelines established by the Animal Care Committee of the University of Bonn Medical Centre. In line with the ARRIVE guidelines, every effort was made to minimize animal pain and distress, as well as to reduce the number of animals used. Animals had unrestricted access to food and water, were provided with nesting material, and were housed under a 12:12 h light/dark cycle, at a controlled temperature of 22 ± 2 °C and relative humidity of 55 ± 10%.

### Manufacturing of injection-pipettes

Custom-made pipettes were prepared as previously detailed¹. In brief, GB150F-8P glass capillaries were pulled using a P87 horizontal puller (Sutter Instruments). The tips were then beveled at a 45° angle using a 1300M Micropipette Beveller (World Precision Instruments). Each pipette was screened on a MF-900 microforge (Narishige, and only those with debris-free tips and openings smaller than 3 μm were selected for use in experiments. After beveling, pipettes were stored for up to 4 weeks in air-tight containers.

### Preparation of acute brain slices

Mice were anesthetized via inhalation of isoflurane and subsequently decapitated. Brains were rapidly extracted and immersed in ice-cold modified artificial cerebrospinal fluid (mACSF), continuously bubbled with 95% O_2_/5% CO_2_. Horizontal cortical acute brain slices (300- or 500-µm thick) were prepared using an HM650 V vibratome (Thermo Fisher Scientific). Following slicing, tissue sections were incubated in mACSF at 35 °C for 30 min and then transferred to standard artificial cerebrospinal fluid (sACSF), also saturated with 95% O_2_/5% CO_2_. Throughout all experiments, slices were maintained at room temperature in oxygenated sACSF at a stable pH of 7.4 and were used within 6–8 h of preparation, allowing for internally consistent comparisons across developmental stages.

For embryonic brain slice preparation, pregnant dams were anesthetized and decapitated. Embryos were immediately extracted, decapitated, and their brains rapidly isolated into ice-cold mACSF following a published protocol [25] with some modifications. To facilitate slicing of embryonic and neonatal tissue, brains were embedded in 4% agarose (Super LM, Carl Roth) prior to sectioning with the vibratome.

### Experimental setup and intravascular perfusion

The ISMICAP experimental setup was used as previously described [4, 5]. Briefly, brain slices were placed in a submerged recording chamber continuously perfused with oxygenated sACSF, and imaged using a two-photon laser scanning microscope (2PIMS-PMT−20, Scientifica) equipped with a Chameleon Vision II laser (Coherent) and operated via ScanImage r3.8 software. For each experiment, laser wavelength, power, and detector gain settings were individually optimized according to the properties of the fluorophore used. Within each slice, thin-walled venules measuring 30–50 μm in diameter were visually identified and selected for impalement. Perfusion pipettes, each filled with 5 µl of the test fluorophore, were mounted on a micromanipulator (Luigs & Neumann) and connected to a pressure pump (ALA Scientific Instruments). Following the impalement of the venular wall, a constant pressure of 100 mmHg was applied and maintained throughout the duration of the experiment. Due to the small diameter of the injection pipette tip (~ 2 μm) and the resulting high hydraulic resistance, a substantial pressure drop occurs across the pipette tip; accordingly, the applied pressure represents an upstream device setting and does not correspond to the hydrostatic pressure within the perfused venule. In this study, different molecular probes were perfused into vasculature, whose respective physicochemical and imaging properties are listed in Table 1.

### Biocytin tetramethylrhodamine assay

Biocytin-tetramethylrhodamine (TMR, Thermo Fisher) was prepared at a concentration of 100 µM in sACSF and perfused into cortical or striatal vessels. Z-stack images of capillaries were acquired 30 min after initiating pressure application, with a plane spacing of 1 μm, at λ_ex_ 820 nm. The distance from the impalement site to the imaged capillaries ranged between 100 and 120 μm. Using ImageJ [26], fluorescence intensity line profiles were extracted from regions of interest (ROIs) oriented perpendicular to the capillaries and normalized to the maximum intraluminal fluorescence. To evaluate BBB permeability, extravascular fluorescence was quantified within a 10 μm region located 3 μm away from both sides of the capillaries at the 30-min time point. These values were normalized and averaged. All fluorescence measurements were corrected for background signal.

### Sulforhodamine 101 assay

Sulforhodamine 101 (SR101, Biomol) was diluted in sACSF to a final concentration of 400 µM and perfused into cortical or striatal vasculature as described above. Z-stacks of capillaries were acquired 30 min after dye application at λ_ex_ 760 nm. Images were background-corrected and analyzed as previously detailed.

### 7-hydroxycoumarin−3-carboxylic acid assay

7-hydroxycoumarin−3-carboxylic acid (7HCC, Sigma-Aldrich) was prepared at a working concentration of 1 mM in sACSF and perfused as detailed. Z-stack imaging of capillaries was performed after 30 min at 760 nm. Acquired images were corrected for background and processed according to the previously established analysis pipeline. In another set of experiments, 7HCC and SR101 were co-perfused for 30 min, with their individual working concentrations maintained at 1 mM and 400 µM, respectively. Image acquisition was carried out at 760 nm to assess colocalization within capillaries.

### FM1-43 assay

FM1−43 (Thermo Fisher Scientific) was diluted in sACSF to a final concentration of 40 µM and perfused for 30 min. Z-stacks and single plane acquisitions of capillaries were obtained at λ_ex_ 840 nm. Fluorescence intensity line profiles were generated as previously described, with abluminal signals normalized to their corresponding luminal fluorescence for each EC. All fluorescence measurements were corrected for background.

### Pericyte counting and live-labeling with Neurotrace 500/525

Neurotrace 500/525 (NT500/525, Thermo Fisher Scientific), a live-cell tracer specific for PCs, was used to label PCs in acute brain slices, following a previously published protocol [27] with minor modifications [5]. Briefly, slices were incubated with NT500/525 (1:250) for 5 min, then rinsed with sACSF. To enable visualization of PCs deeper within the tissue, 50-µm z-stacks were acquired 45 min post-incubation at λ_ex_ 760 nm. In some recordings, the laser-Dodt contrast channel was acquired in parallel with the fluorescence signal to aid in cellular visualization.

### Bovine serum albumin assay

BSA-Alexa Fluor 488 conjugate (66.5 kDa, Thermo Fisher) was prepared to a final concentration of 25 µg/ml in sACSF and perfused for 30 min in cortical vasculature following the procedure described above. Subsequently, z-stack images of capillaries were acquired at λ_ex_ 760 nm. Intensity line profiles were extracted from ROIs positioned perpendicular to the capillary axis and normalized to the maximum fluorescence within the vascular lumen.

**Table 1:** Physicochemical and imaging properties of the fluorescent tracers used in the study

Tracer	MW (Da)	Polarity/charge at physiological pH	One-photon λ_ex_ (nm)	One-photon λ_em_ (nm)	Two-photon λ_ex_ (nm) used in the study	Supplier	Catalog number
Biocytin-TMR	869	Polar, net charge depends on pH	554	581	820	Thermo Fisher	T12921
SR101	607	Anionic	586	605	760	Biomol	Cay16953−50
7HCC	206	Predominantly anionic	342 (pH 4) 386 (pH 9)	447 (pH 4) 448 (pH 9)	760	Sigma Aldrich	55155
FM1−43	611	Cationic, amphipathic styryl dye; membrane-associated	510	626	840	Thermo Fisher	T3163
BSA-Alexa Fluor 488	66,000	Anionic	495	519	760	Thermo Fisher	A13100

### Data processing and statistical analysis

Unless stated otherwise, all images represent raw data without the application of denoising or smoothing algorithms. For enhanced visualization, various ImageJ look-up tables (LUTs) - including red, magenta, blue, green, yellow, and royal - were applied where appropriate. The ‘royal’ LUT is a color scale that maps low-intensity signals to blue and high-intensity signals to white. Data are expressed as mean ± standard error of the means (SEM), unless stated otherwise. For spatial distribution analyses, values are reported as mean ± SD together with median and interquartile range (IQR) as descriptive measures of the spread and distribution of measured values. The number of analyzed regions of interest (n_ROI_) is indicated in the respective figure legends. Statistical analysis was conducted using one-way ANOVA. Unless stated otherwise, Tukey’s post *hoc* test was applied to correct for multiple pairwise comparisons. For comparisons between successive developmental stages in selected time-course analyses, Holm-Sidak multiple-comparison test was used as indicated in the respective figure legends. A P-value < 0.05 was considered indicative of statistical significance. Adjusted pairwise comparisons were two-sided. All statistical tests were performed using GraphPad Prism version 9.0.0 (GraphPad Software, CA, USA).

## Results

### Permeability to small-molecule probes in striatum transiently increases in neonates

TJ normally limit the passive diffusion of molecules through the intercellular cleft between adjacent ECs. Thus, intravascular perfusion of a fluorescent tracer and quantification of its extravascular signal around capillaries provides a measure of barrier permeability [5]. In this study, we adapted this concept to assess the barrier integrity in striatum during development, namely in embryos (E18), neonates (P0-P2), early postnatal pups (P12) and adolescent mice (P25).

**Fig. 1:**
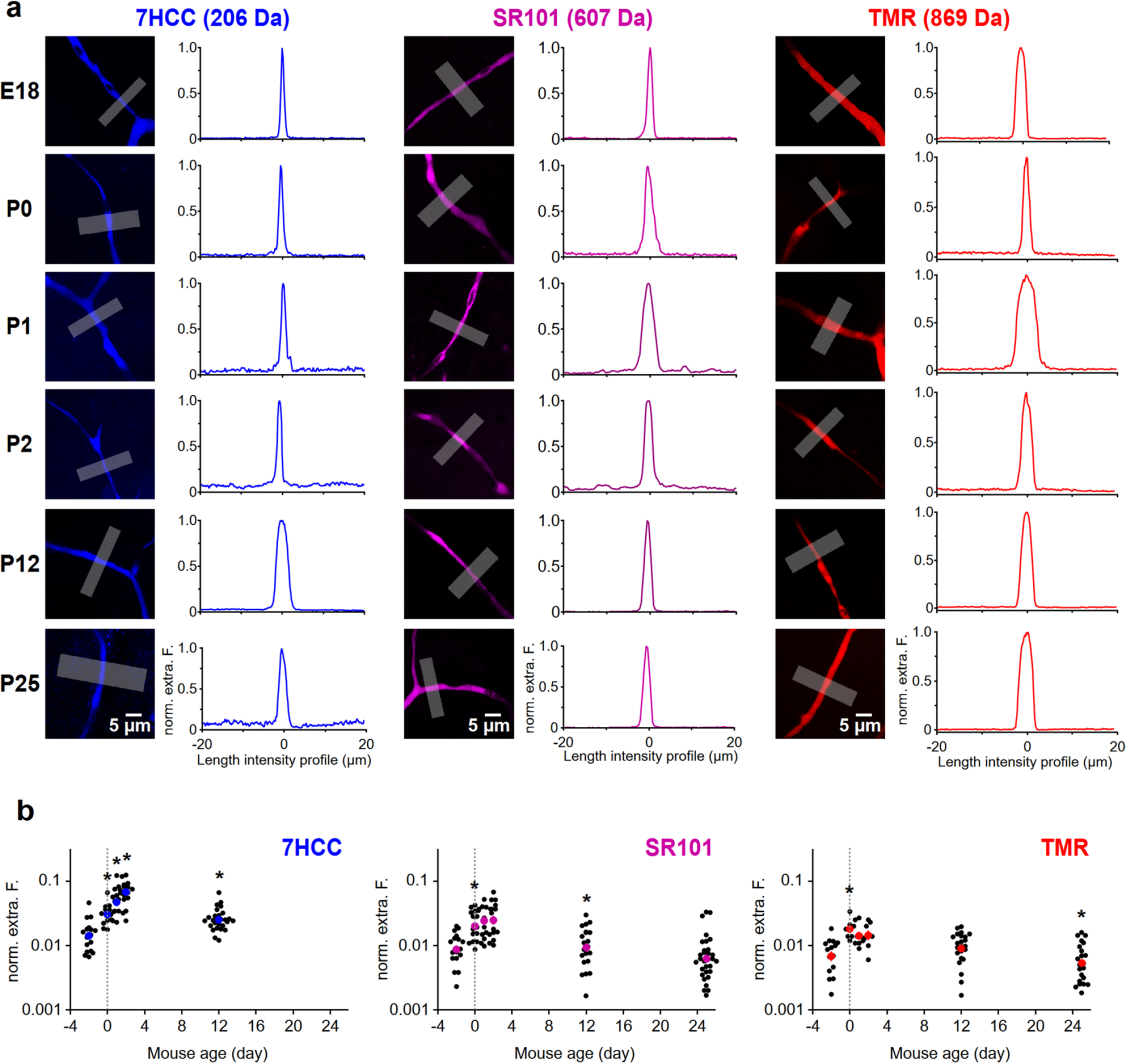
Small-molecule tracer diffusion in the striatum increases abruptly but transiently in neonates. **a**, Single frames of striatal capillaries, 100–120 μm away from the impalement point, in embryonic (E18), neonatal (P0, P1, P2), early postnatal (P12) and adolescent (P25) mouse brains 30 min post perfusion of three probes (7HCC, SR101 and TMR) independently and the corresponding spatial fluorescence of a line profile (grey) normalized to the intravascular fluorescence. The analyzed line profiles were within 10 μm in a 3-µm distance to the sides of capillaries. **b**, Quantification of extravascular fluorescence intensities within the specified ROIs across capillaries at 30 min. Data are represented as mean ± SEM, and individual data points are overlaid. All intensities were background-corrected and normalized to the intravascular fluorescence. The vertical dotted line indicates delivery/birth. Data were obtained from 20 injections/19 embryos, 31 injections/30 neonates, 11 injections/6 early postnatal mice, and 19 injections/9 adolescent mice (n_ROI_ E18 = 49, n_ROI_ P0 = 36, n_ROI_ P1 = 33, n_ROI_ P2 = 39, n_ROI_ P12 = 72, n_ROI_ P25 = 58). For each tracer, statistical analysis was performed on log-transformed values using one-way ANOVA followed by Holm-Sidak multiple-comparison testing between successive developmental stages only (*P < 0.05). Asterisks indicate significant differences compared with the immediately preceding developmental stage

To this end, we applied ISMICAP combined with two-photon microscopy and acute brain slices. In this setup, individual striatal venules (30–50 μm diameter) were impaled with glass pipettes containing fluorescent tracers and perfused under a constant intraluminal pressure of 100 mmHg, while tracer diffusion across the vascular wall was monitored in real time. Three small-molecule probes, 7HCC, SR101 and TMR, were independently perfused into striatal venules. After 30 min, the extravascular fluorescence was quantified within 10-µm segments located 3 μm on either side of capillaries using line profiles (Fig. 1a). At embryonic stage (E18), all three probes showed low levels of extravascular diffusion of 1.6 ± 0.3%, 1.0 ± 0.1% and 0.8 ± 0.2% of the intraluminal fluorescence for 7HCC, SR101 and TMR, respectively (Fig. 1b). Successive-stage analysis of log-transformed values revealed a significant increase in extravascular fluorescence during the early neonatal period, consistent with an abrupt rise in small-molecule tracer permeability after birth at P0 (Fig. 1b). At later stages, extravascular fluorescence decreased progressively, indicating restoration of a more restrictive barrier phenotype by early postnatal and adolescent stages. For SR101 and TMR, the extravascular fluorescence reached the lowest levels at P25 (0.9 ± 0.2% and 0.7 ± 0.4% of intraluminal fluorescence, respectively; Fig. 1b).

For 7HCC, one dataset (P25) could not be acquired because the dye signal was extremely weak during two-photon imaging of the developed striatum (excitation at 760 nm; detection with 472/30 nm bandpass filter). Fluorescence was detectable only in large venules and surface capillaries, whereas signal intensity decreased markedly with imaging depth and capillary-level detail was largely lost due to a low signal-to-noise ratio. This attenuation may reflect the optical properties of striatal tissue, particularly the dense axonal fiber arrangement, which is known to enhance light scattering [28]. Although using longer excitation wavelengths can mitigate scattering, 7HCC fluorescence was only detectable at λ_ex_ 760 nm. Since 7HCC has not been widely applied in brain tissue and its spectroscopic properties remain poorly characterized, no reference data are currently available to explain this effect.

In conclusion, BBB permeability to small-molecule tracers in the striatum does not follow a simple linear tightening process during development, but instead includes a transient permeability window before re-establishing a more restrictive postnatal/adolescent barrier phenotype.

### Small-molecule probes accumulate in distinct perivascular and endothelial compartments in neonates

Here, we investigated the cellular fate of diffused small-molecule probes during the neonatal period of transiently increased BBB permeability (P0 - P2 pups) in striatum.

**Fig. 2:**
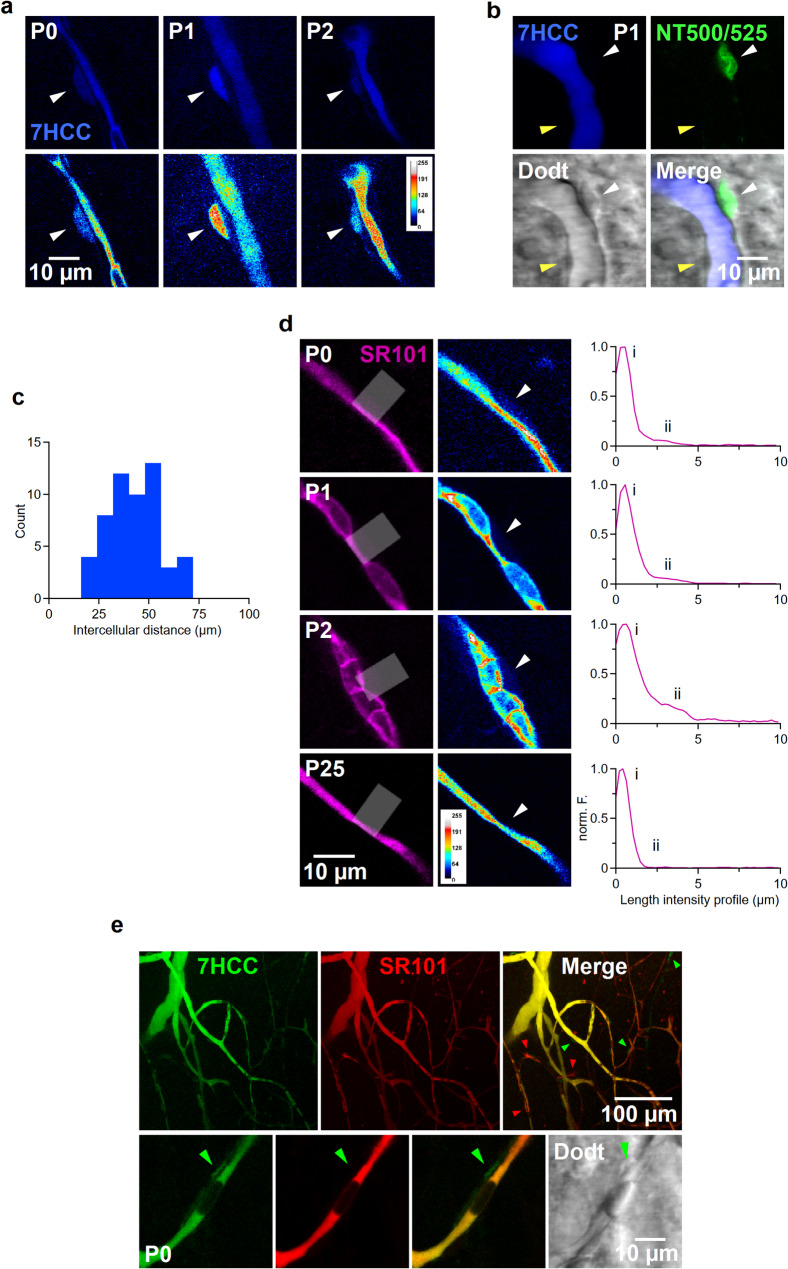
Perivascular cells accumulate small-molecule tracers in neonates. **a**, Single frames of striatal capillaries in neonates (P0, P1, P2) 30 min post perfusion of 7HCC showing the accumulation of the dye into unidentified perivascular cells (arrowheads). The ‘royal’ LUT color map encodes pixel intensity, with ‘blue’ indicating low values and ‘white’ indicating high values. Images are representative of 5 experiments. **b**, Single frame of a capillary in P1, where PCs were prelabelled with NT500/525 (white arrowheads) and 7HCC was perfused for 30 min. The laser Dodt scan shows an EC (yellow arrowheads). The recorded individual channels show no overlap between NT500/525 and 7HCC. **c**, Histogram of intercellular distances between perivascular 7HCC-positive cells along vessels. Distances are reported descriptively as sample mean ± SD and median [IQR]. The mean distance was 45.4 ± 15.0 μm, with a median of 46.1 μm [IQR: 33.1–55.4 μm]. Data were obtained from 16 injections/15 neonates (*n* = 54). **d**, Single frames of striatal capillaries in neonates and P25 mice perfused with SR101 (left) for 30 min, also shown using ‘royal’ LUT, and the corresponding spatial fluorescence (right) of line profiles (grey) across the capillary wall normalized to the intravascular fluorescence, where ‘ii’ peak corresponds to the cytoplasmic accumulation of SR101 in ECs in neonatal capillaries (arrowheads), whereas comparable SR101 accumulation was not observed at P25. Images are representative of 5 experiments. **e**, MIPs of venules (top) and a single frame of a capillary (bottom) in P0, perfused simultaneously with 7HCC and SR101 for 30 min. Both dyes colocalize within vessels, yet distinct structures were labeled exclusively by 7HCC (green arrowheads) or SR101 (red arrowheads), suggesting differential dye uptake or transport. Single-channel images and the overlay are shown for venules, whereas for the capillary the corresponding Dodt scan is additionally provided. Gamma adjustment was applied to the green and red channels to enhance the visibility of weaker structures. Images are representative of 4 experiments

Perfusion of 7HCC for 30 min revealed a striking accumulation of the dye within unidentified spindle-shaped perivascular cellular structures along capillaries, positioned slightly away from the endothelial wall, with a mean gap of 2.8 ± 0.3 μm (Fig. 2a). To assess whether these cells corresponded to PCs, NT500/525 was used to label PCs prior to the intravascular perfusion of 7HCC. The fluorescent channels showed no detectable overlap between NT500/525-labeled PCs and 7HCC signal, suggesting that the 7HCC^+^ cells represent a distinct perivascular population from NT500/525-labeled PCs (Fig. 2b, white arrowheads). In the same recordings, 7HCC^+^ cell bodies were spatially separated from the endothelial wall, arguing against an endothelial localization (Fig. 2b, yellow arrowheads). Quantitative analysis of the spatial distribution of these 7HCC^+^ perivascular cells along vessels showed an intercellular distance of 45.4 ± 15.0 μm, with a median distance of 46.05 μm [IQR: 33.1–55.4 μm] (*n* = 54) (Fig. 2c).

SR101 displayed a different pattern. In neonates, SR101 accumulated within the cytoplasm of ECs (Fig. 2d), as demonstrated by line profiles in which the first fluorescent peak (i) corresponded to the intravascular fluorescence and the secondary peak (ii) to cytoplasmic uptake. In contrast, comparable cytoplasmic SR101 accumulation was not observed at P25. Interestingly, when 7HCC and SR101 were perfused simultaneously in P0 striata, both dyes colocalized within venules and most capillaries. However, sole labeling of distinct vascular and perivascular structures by either probe was also observed throughout the vascular tree (Fig. 2e). Structures positive only for 7HCC or only for SR101 were consistently detected. To enhance the visibility of weaker signals, gamma adjustment was applied to the green and red channels. While single-channel and overlay images are shown for venules, the corresponding Dodt scan is additionally provided for the capillary example, where a filamentous perivascular structure appeared to be labelled solely by 7HCC (Fig. 2e, bottom).

Together, these findings indicate that during the neonatal permeability window, small-molecule probes displayed distinct vascular and perivascular fluorescence patterns, including endothelial accumulation of SR101 and selective labeling of 7HCC^+^ perivascular cells. These observations suggest tracer-specific vascular/perivascular handling rather than uniform passive diffusion into the parenchyma.

### FM1−43 membrane probe exhibits increased abluminal membrane diffusion and pericyte uptake in neonates

We next examined the membrane permeability of ECs in striata during development using FM1-43, a styryl dye that intercalates into endothelial membranes and diffuses laterally from the luminal to the abluminal side. TJ comprise intramembranous domains, embedded in the lipid bilayer and restricting diffusion along membranes, as well as extramembranous domains that span neighboring ECs to seal the paracellular cleft [4, 5]. In the mature BBB, FM1-43 predominantly labels luminal membranes and only slowly diffuses to abluminal membranes. Thus, the rate and extent of abluminal FM1-43-associated fluorescence provide a functional readout of membrane-associated dye access/diffusion across the endothelial barrier, which may reflect changes in intramembranous junctional restriction but does not by itself exclude other developmental changes in membrane accessibility or tracer accumulation.

**Fig. 3:**
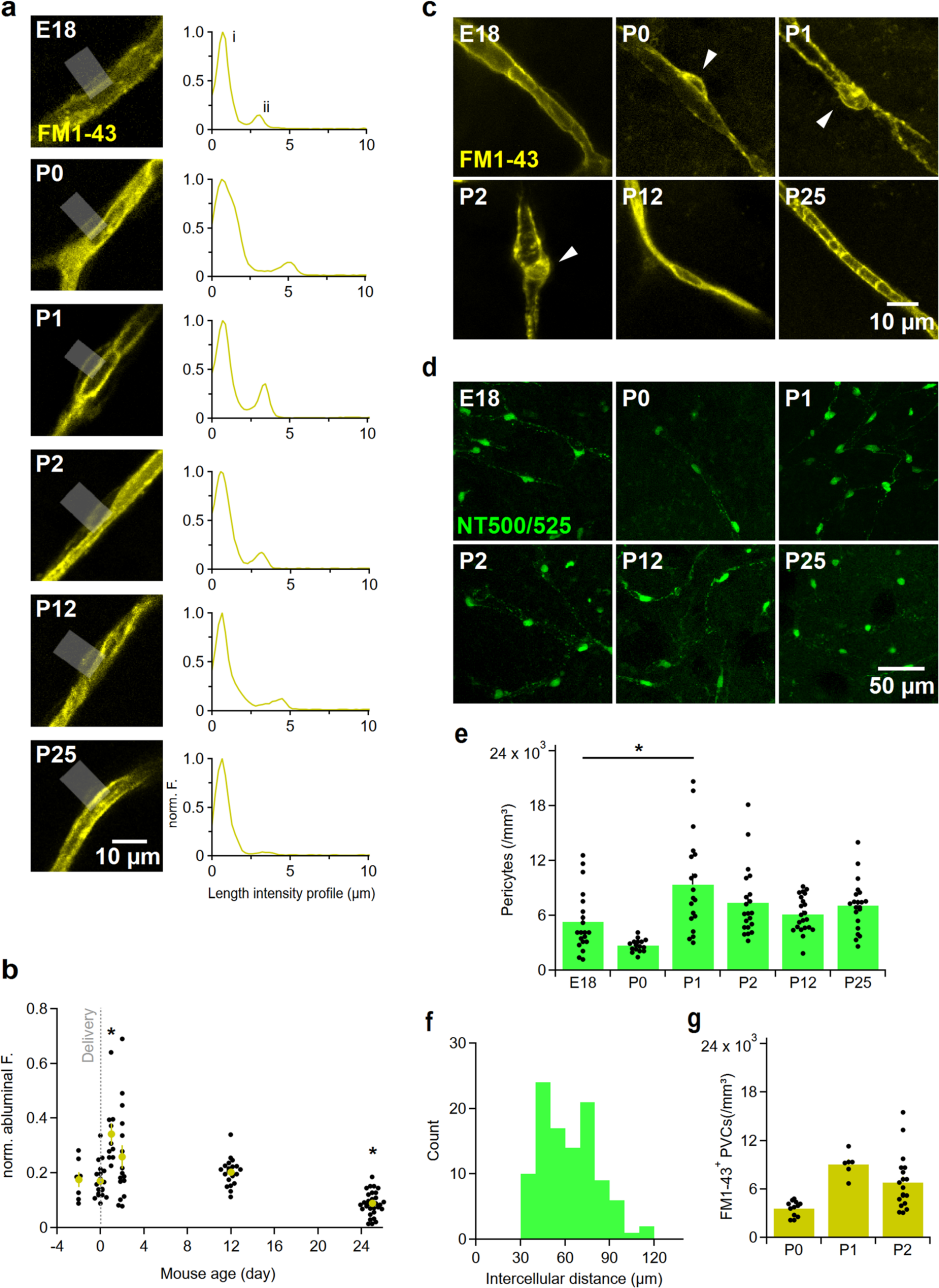
FM1-43 shows increased abluminal membrane-associated fluorescence in neonatal endothelial cells and accumulates in pericytes. **a**, Single frames of striatal capillaries in embryonic (E18), neonatal (P0, P1, P2), early postnatal (P12) and adolescent (P25) mouse brains 30 min post perfusion of FM1-43 and the corresponding spatial fluorescence profiles normalized to the luminal fluorescence, where ‘i’ peak corresponds to the luminal fluorescence, while ‘ii’ peak corresponds to the abluminal fluorescence. **b**, Quantification of extravascular fluorescence intensities within the specified ROIs across capillaries. Data are represented as mean ± SEM, and individual data points are overlaid. All intensities were background corrected and normalized to the luminal fluorescence. Data were obtained from 9 injections/8 embryos, 17 injections/15 neonates, 4 injections/3 early postnatal mice, and 5 injections/3 adolescent mice (n_ROI_ E18 = 7, n_ROI_ P0 = 20, n_ROI_ P1 = 10, n_ROI_ P2 = 16, n_ROI_ P12 = 21, n_ROI_ P25 = 31). Statistical analysis was performed using one-way ANOVA followed by Holm-Sidak multiple-comparison testing between successive developmental stages only (*P < 0.05). Asterisks indicate significant differences compared with the immediately preceding developmental stage. **c**, MIPs of striatal capillaries demonstrating the diffusion of FM1-43 into perivascular cells (arrowheads) in neonates, which was absent at other developmental stages. The images are representative of 5 experiments. **d**, MIPs of 50-µm stacks of striatal tissue across all developmental stages, with PCs were prelabelled using NT500/525. Images are representative of 8 independent experiments. **e**, Pericyte quantification in striata of embryos, neonates, early postnatal and adolescent mice. At P1, PC count was 1.8-fold higher than at E18 (one-way ANOVA followed by Tukey’s multiple-comparison test, *P < 0.05). Data were obtained from 8 experiments (n_ROI_ E18 = 20, n_ROI_ P0 = 16, n_ROI_ P1 = 19, n_ROI_ P2 = 21, n_ROI_ P12 = 22, n_ROI_ P25 = 21). **f**, Histogram of intercellular distances between NT500/525-labeled pericytes along vessels in P1 striata. Distances are reported descriptively as sample mean ± SD and median [IQR]. The mean intercellular distance was 60.8 ± 22.8 μm, with a median of 55.7 μm [IQR: 42.9–75.1 μm] (*n* = 118). **g**, Quantification of FM1-43^+^ perivascular cells in neonatal striata. Statistical analysis was performed using one-way ANOVA followed by Tukey’s multiple-comparison test (*P < 0.05). Data were obtained from 5 experiments (n_ROI_ P0 = 16, n_ROI_ P1 = 6, n_ROI_ P2 = 19)

In embryonic striata (E18), line profiles across ECs revealed that FM1-43 predominantly labelled luminal membranes, while a small, yet measurable, abluminal signal was detected after 30-min perfusion (Fig. 3a). In contrast, neonatal stages showed a significant increase in abluminal FM1-43 diffusion, consistent with the transient increase in BBB permeability identified in this developmental window. Quantification of normalized abluminal fluorescence showed that abluminal FM1-43 at P0 was comparable to that at E18, followed by a significant nearly 2-fold increase from P0 to P1, where the signal reached its peak (*P < 0.05, Fig. 3b). The increased neonatal signal was transient, with fluorescence decreasing at later stages and reaching its lowest levels at P25 (0.09 ± 0.01). Successive-stage comparisons showed a significant decrease from P12 to P25, consistent with progressive restoration of barrier restriction during postnatal maturation (Fig. 3b). Although the FM1-43 signal appeared membrane-associated, developmental changes in membrane accessibility or tracer accumulation cannot be excluded.

Beyond abluminal diffusion in ECs, we noticed that FM1-43 labelled distinct perivascular cells during the neonatal period. MIPs at P0-P2 revealed FM1-43^+^ somata protruding from the capillary wall (arrowheads), which displayed morphology consistent with PCs: compact ovoid somas tightly apposed to capillaries, with fine longitudinal processes extending along the vascular axis (Fig. 3c). Their stereotypic spacing and perivascular localization supported a PC-like/mural localization. Since FM1-43 and NT500/525 are both detected in the same spectral channel, direct co-labeling was not feasible. Instead, we pre-labelled PCs with NT500/525 across developmental stages in another set of experiments. MIPs of 50-µm stacks confirmed the presence of PCs at all ages (Fig. 3d), with counts revealing a transient peak at P1, being 1.8-fold higher than at E18 (one-way ANOVA, followed by Tukey’s multiple-comparison test, *P* < 0.05; Fig. 3e). Importantly, this peak in PC number coincided with the phase showing maximal FM1-43 abluminal diffusion. Descriptive spatial analysis of NT500/525-labeled PCs at P1 showed an intercellular distance of 60.8 ± 22.8 μm, with a median distance of 55.71 μm [IQR: 42.93–75.13 μm] along vessels (*n* = 118; Fig. 3f). To directly compare the temporal dynamics of perivascular FM1-43 uptake with the abundance of PCs, we quantified FM1-43^+^ perivascular cells across neonatal stages. FM1-43^+^ cell density was significantly higher at P1 and P2 compared to P0, with the highest mean value observed at P1, although P1 and P2 were not significantly different from each other (Fig. 3g). This temporal profile was similar to that observed for NT500/525-labeled PCs (Fig. 3e). Together with the absence of a detectable gap from the endothelium and the shared basement-membrane localization, this pattern is consistent with the interpretation that FM1-43^+^ perivascular cells are consistent with PCs/PC-like mural cells. This spatial and temporal profile differed from that of the 7HCC^+^ perivascular cells (Fig. 2c), arguing against the 7HCC^+^ population representing typical PCs.

Taken together, these findings indicate that during the neonatal permeability window, FM1-43 diffusion to abluminal endothelial membranes is markedly enhanced and is accompanied by labeling of perivascular/mural cells with features consistent with PCs. The temporal coincidence between peak FM1-43 abluminal diffusion and increased NT500/525-labeled PC counts suggests dynamic remodeling of the NVU during early postnatal development, although definitive cellular identity will require marker-based validation.

### Parenchymal diffusion of bovine serum albumin in neonatal striata is as restricted as in adolescent mice

**Fig. 4:**
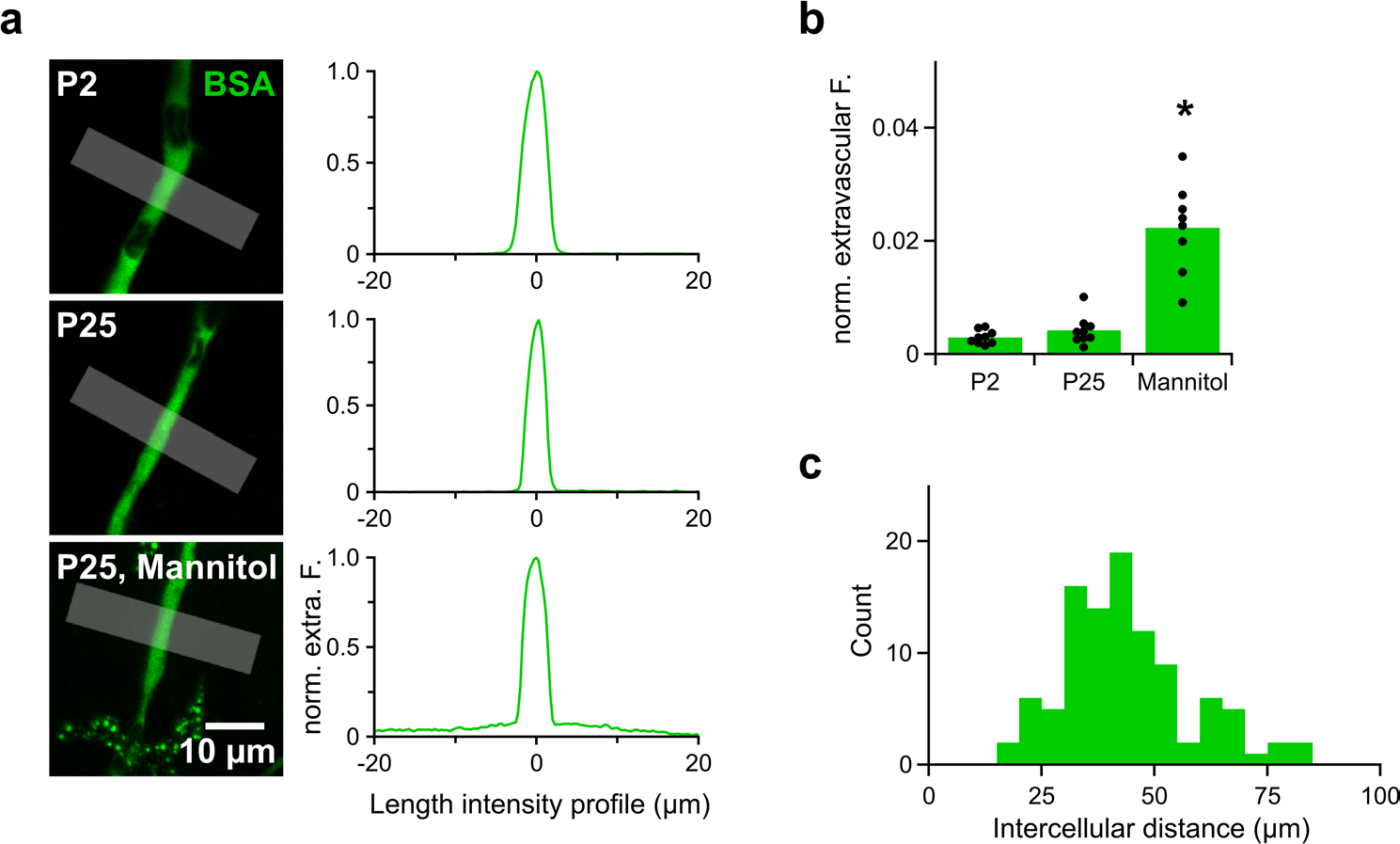
Parenchymal diffusion of bovine serum albumin in neonatal striata is comparable to that in adolescent mice. **a**, Single frames of striatal capillaries in P2 and P25 pups 30 min after perfusion of BSA-Alexa488 (BSA) as well as in P25 pups perfused with BSA in 15% mannitol (left), and the corresponding spatial fluorescence profiles (right) normalized to intravascular fluorescence. Profiles were analyzed within 10 μm, positioned 3 μm lateral to the capillary walls. Images are representative of 6 experiments. **b**, The extravascular fluorescence of BSA normalized to luminal signal in P2 striata was comparable to that in P25 striata. Perfusion with hyperosmolar mannitol for 30 min significantly increased extravascular BSA fluorescence (one-way ANOVA followed by Tukey’s pairwise comparison, *P < 0.05). Data were obtained from 13 injections/ 6 pups (n_ROI_ P2 = 9, n_ROI_ P25 = 9, n_ROI_ P25 mannitol = 8). **c**, Histogram of intercellular distances between perivascular macrophages along microvessels in P25 mice perfused with BSA in 15% mannitol. Distances are reported descriptively as sample mean ± SD and median [IQR]. The mean intercellular distance was 44.9 ± 13.8 μm, with a median of 42.5 μm [IQR: 34.6–51.1 μm] (*n* = 155)

To test whether the transient increase in permeability in neonatal striata observed for small-molecule probes extended to larger proteins, we perfused BSA into striatal venules at P2 and P25 for 30 min. P2 was selected for comparison as it showed the maximum permeability to small-molecule probes. Single frames and corresponding line profiles revealed that extravascular BSA fluorescence was minimal at both stages, with comparable diffusion into the parenchyma (Fig. 4a). Quantification confirmed that extravascular BSA signal normalized to intraluminal fluorescence did not differ between P2 and P25 striata (Fig. 4b), indicating that BSA-sized macromolecular permeability is low at P2 and comparable to P25 under the present ISMICAP setup.

In contrast, hyperosmolar mannitol, which transiently opens TJ through a combination of vasodilation and endothelial shrinkage [29], produced a significant increase in extravascular BSA fluorescence at P25 (one-way ANOVA followed by Tukey’s pairwise comparison, *P* < 0.05; Fig. 4a, b). Within 30 min, clusters of BSA became evident along microvessels, reflecting uptake into perivascular cells [5]. The morphology and distribution of these cells were consistent with perivascular macrophages, which are known to reside in the perivascular space of microvessels and phagocytose macromolecules [30]. Descriptive analysis of their spatial showed intercellular distances of 44.9 ± 13.8 μm, with a median distance of 42.50 μm [IQR: 34.60–51.10 μm] along microvessels (*n* = 155; Fig. 4c), corresponding to an average density of 4.4 × 10^3^ cells/mm^2^.Together, these results indicate that the increased neonatal permeability observed for small-molecule tracers at P2 does not extend detectably to BSA-sized macromolecules under the present experimental conditions. Leakage and cellular uptake of BSA were only observed upon mannitol-induced BBB disruption, supporting a size-restricted permeability phenotype rather than generalized macromolecular leakage.

### Striatal and cortical microvasculature show different permeabilities for small-molecule tracers

**Fig. 5:**
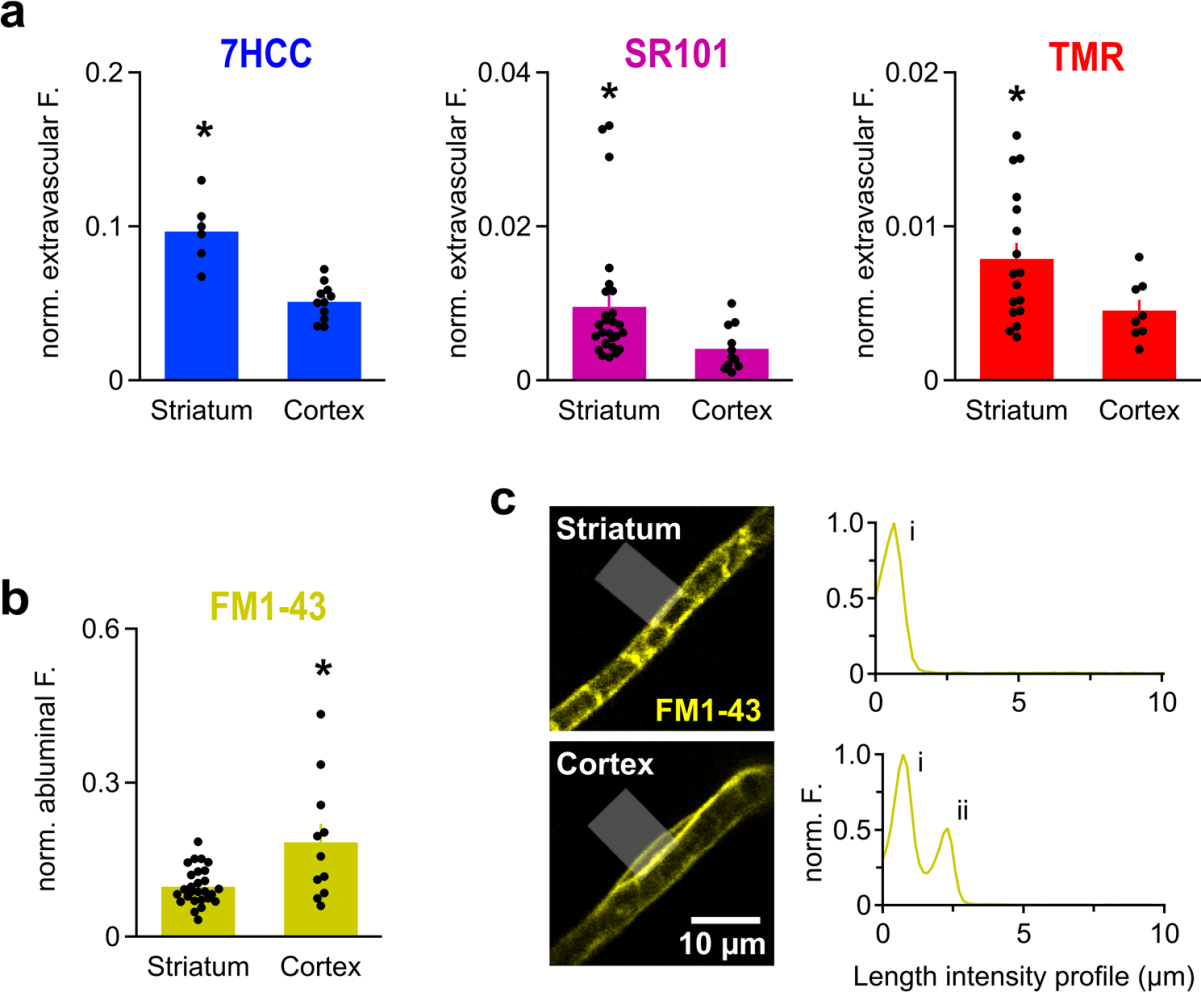
Regional differences in tracer-specific blood-brain barrier permeability profiles between striatum and cortex in adolescent mice. **a**, Quantification of extravascular fluorescence of 7HCC, SR101, and TMR in striata versus cortices of P25 pups. Extravascular fluorescence of 7HCC, SR101, and TMR was significantly higher in striata compared to cortices (Student’s *t*-test, *P < 0.05). Data are represented as mean ± SEM, and individual data points are overlaid. All intensities were background corrected. Data were obtained from 51 injections/23 pups. **b**, Quantification of abluminal fluorescence of FM1-43 in striata versus cortices of P25 pups. FM1-43 revealed higher abluminal fluorescence in cortices than in striatal capillaries (Student’s *t*-test, *P < 0.05). Data are represented as mean ± SEM, and individual data points are overlaid. All intensities were background corrected. Data were obtained from 10 injections/7 pups. **c**, Representative single-frame images of striatal (top) and cortical (bottom) capillaries in P25 mouse brains 30 min after FM1-43 perfusion, shown together with normalized line profiles. Peak ‘i’ denotes luminal fluorescence, whereas peak ‘ii’ corresponds to the abluminal fluorescence

Finally, we assessed whether BBB tracer permeability varies between brain regions by comparing the striatum and cortex of P25 mice. Normalized extravascular fluorescence of the small-molecule probes 7HCC, SR101, and TMR was significantly higher in the striatum than in the cortex, indicating increased tracer permeability in the striatal microvasculature at this stage (Student’s *t*-test, *P < 0.05; Fig. 5a). In contrast, abluminal FM1-43-associated fluorescence was significantly lower in the striatum than in the cortex, revealing an opposite pattern for this membrane-associated probe (Fig. 5b). Consistent with these quantifications, two-photon single-plane images and corresponding spatial intensity profiles (Fig. 5c) illustrate that cortical capillaries display a more prominent abluminal FM1-43 peak compared to striatal vessels.

Together, these findings highlight region-dependent differences in tracer-specific permeability profiles at P25. While small-molecule diffusion was more pronounced in the striatum, membrane-associated FM1-43 fluorescence was greater in the cortex, suggesting that distinct barrier-associated or tracer-handling processes differ between regions at this developmental stage. These tracers likely report distinct aspects of barrier function, including extravascular small-molecule access, endothelial or perivascular uptake, and membrane-associated processes.

## Discussion

**Fig. 6:**
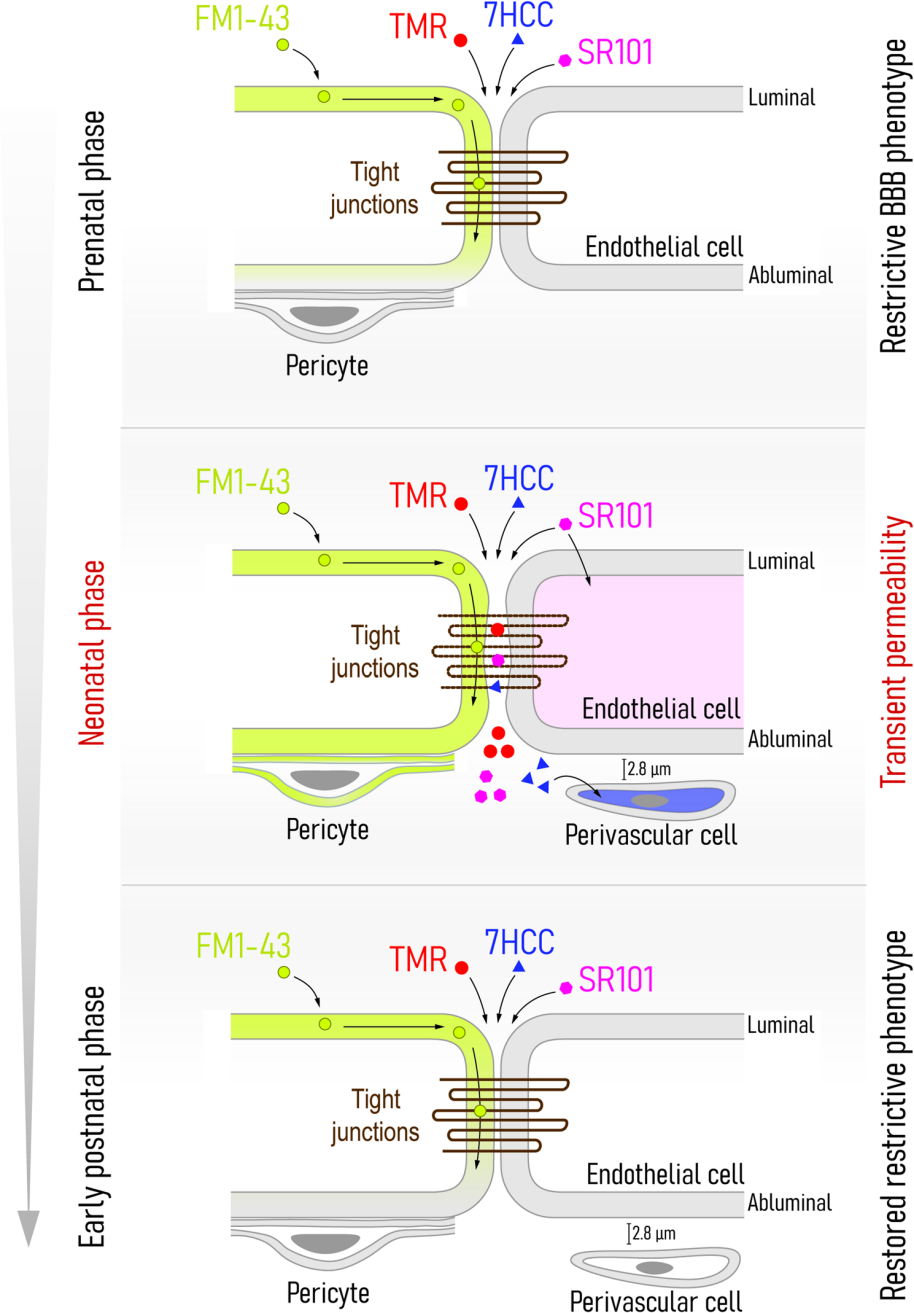
Schematic representation of the developmental dynamics of tracer-specific blood-brain barrier permeability in the striatum. BBB maturation in the striatum follows a non-linear course. In the prenatal phase (E18, top panel), the BBB is already functionally restrictive, limiting detectable extravascular access of the small-molecule tracers TMR, 7HCC, and SR101, as well as abluminal FM1-43 diffusion. In the neonatal phase (P0-P2, middle panel), a transient permeability window emerges, characterized by increased diffusion of small-molecule diffusion, accumulation of 7HCC in perivascular cells, accumulation of SR101 in endothelial cytoplasm, and labelling of abluminal endothelial membranes and pericytes by FM1-43. During the early postnatal/adolescent phase (P12-P25, bottom panel), tracer permeability is reduced again, consistent with restoration of a more restrictive BBB phenotype. The schematic summarizes tracer-specific fluorescence patterns and does not imply that the observed signals exclusively reflect TJ-mediated permeability

In this study, we systematically characterized the developmental dynamics of small-molecule permeability across the BBB in the mouse striatum using the ISMICAP approach. We found that the BBB was already functionally restrictive at late embryonic stage (E18), indicating that key barrier properties are established prenatally. Importantly, small-molecule tracer permeability increased transiently during the neonatal phase (P0-P2), followed by progressive normalization by P12 and further restriction into adolescence (Fig. 6). This transient permeability window was observed with tracers reporting distinct vascular, abluminal, and perivascular fluorescence patterns despite morphologically intact vasculature. Together, these findings reveal that BBB maturation in the striatum does not proceed simply through monotonic progressive restriction, but instead undergoes a distinct perinatal phase of altered tracer-specific barrier function.

The tracers used in this study were selected to provide complementary functional readouts based on their molecular weight, polarity, and vascular/perivascular labeling behavior. TMR-biocytin and 7HCC are polar small-molecule tracers and are not established substrates of major BBB efflux transporters. SR101 is a highly water-soluble sulfonic acid tracer with known cellular labeling properties in neural tissue, and was therefore interpreted with particular attention to cellular accumulation rather than as a purely extracellular diffusion marker. FM1-43 was used as a membrane-associated tracer to visualize abluminal access and perivascular/mural cell labeling, whereas BSA served as a macromolecular permeability reference. Thus, the tracer panel was designed to capture tracer-specific BBB access and vascular/perivascular handling under controlled luminal delivery, while recognizing that the present approach does not by itself mechanistically isolate junctional, vesicular, or transporter-mediated routes.

Our findings are consistent with the established timeline of BBB differentiation during embryogenesis. Vascular sprouts invading the neural tube between E12 and E15 already express molecular markers of barrier specialization such as claudin-5 and occludin, and functional tracer studies indicate that the fetal BBB restricts large molecules by late gestation (E18) [9, 10, 31]. This supports the view that the barrier structure is largely established before birth. However, the transient permeability increase observed in our study indicates that barrier function is not static once established but may fluctuate during postnatal maturation.

A plausible explanation is that, during the perinatal transition, the NVU undergoes rapid remodeling, with PCs still recruiting and extending processes, astrocytic endfeet progressively ensheathing vessels, and the basement membrane continuing to mature, as reviewed elsewhere [32]. These dynamic changes may transiently destabilize endothelial junctions by altering mechanical forces, paracrine signaling, or local metabolic balance. In addition, the abrupt physiological shift from placental to pulmonary circulation at birth leads to marked increases in cerebral blood flow, oxygen tension, and reactive oxygen species [33], all of which could potentially modulate TJ phosphorylation or other regulatory processes and temporarily affect junctional adhesion. Finally, efflux transporters such as ATP-binding cassette (ABC) transporter B1 (ABCB1) continue to mature postnatally [34], potentially reducing active efflux capacity and favoring passive diffusion during the early neonatal phase. Together, these mechanisms provide a coherent framework to explain a brief developmental window of increased permeability before the NVU achieves full stabilization. This interpretation aligns with our observation that small-molecule tracer permeability transiently increases postnatally while overall vascular morphology remains intact.

During this transient permeability window in neonates, small-molecule probes, namely 7HCC and SR101, accumulated within distinct vascular and perivascular compartments (Fig. 6). 7HCC (206 Da) preferentially labeled a population of perivascular cells arranged regularly along capillaries, whereas SR101 (607 Da) accumulated in endothelial cytoplasm. The coexistence of distinct tracer-positive compartments suggests that, during this transient barrier-permeability window, diffused solutes are not passively dispersed but selectively sequestered by vascular and perivascular cell populations, possibly contributing to clearance or signaling at the vascular interface. Cerebral ECs have recently been shown to display phagocytic and endocytic activity, capable of internalizing and processing extracellular material [35]. Such uptake may represent developmental sampling of the perivascular environment, membrane turnover, or metabolic recycling during junctional remodeling. Moreover, SR101 is a known substrate of the efflux transporter ABCC2 [36]; thus, reduced ABCC2 activity in neonates would limit extrusion and thereby allow its cytoplasmic retention. A transient decline in transporter activity may therefore reinforce the overall increase in BBB permeability detected at this age.

When 7HCC and SR101 were co-perfused in neonatal striata (P0), the resulting pattern was reproducible across multiple preparations and revealed three distinct labeling domains: (i) vascular lumens showing colocalization of both tracers, (ii) perivascular cells that were labeled exclusively by 7HCC, and (iii) fine filamentous or elongated structures, most of which showed only 7HCC signal, whereas a few vessel-like structures were exclusively labelled by SR101. This consistent partial overlap indicates that the two tracers probe different diffusion and retention routes within ECs. The colocalization intravascularly suggests shared intravascular delivery and overlapping luminal access, whereas the selective 7HCC labeling of perivascular and filamentous compartments suggests preferential access to those compartments. In contrast, SR101, though similar in lipophilicity, is larger in size and more polar, showing limited extravascular diffusion but some endothelial and filamentous labeling. Together, these complementary patterns highlight micro-regional variability in tracer access and retention and tracer diffusion within the neonatal striatum, supporting the concept of a transient, spatially heterogeneous permeability pattern during early postnatal maturation.

FM1-43 experiments revealed a transient increase in abluminal membrane-associated fluorescence that followed the same transient pattern as paracellular diffusion, suggesting that membrane-associated barrier properties are also dynamically regulated during the perinatal period. Notably, the phase of maximal FM1-43 diffusion coincided with a transient rise in PC number as well as FM1-43^+^ perivascular cells. This temporal overlap suggests that PCs may participate in or respond to junctional remodeling during early postnatal development. The observed uptake of FM1-43 by PCs is consistent with their emerging roles in vessel stabilization and debris clearance within the NVU, especially since they share the basal membrane with ECs [32].

In contrast to small-molecule probes, BSA showed no enhanced extravascular diffusion in P2 neonates compared with P25, indicating that the neonatal permeability window detected with small molecules does not extend detectably to BSA-sized macromolecules under the present conditions. However, because E18 was not included in the BSA analysis, these data do not resolve whether BSA permeability differs between late embryonic and neonatal stages. Notably, transcellular vesicular transport is strongly suppressed in cerebral ECs compared to peripheral endothelia, largely through Mfsd2a-dependent inhibition of caveolae formation, which establishes the characteristically low transcytosis rate of the BBB. The absence of BSA extravasation in our experiments is therefore consistent with a barrier state in which macromolecular leakage and vesicular transport remain limited. The absence of BSA extravasation is consistent with a size-restricted barrier alteration rather than generalized macromolecular leakage [37]. Osmotic disruption with mannitol induced BSA leakage and uptake by perivascular macrophages, consistent with our previous marker-based characterization of these cells [5]. Together, these findings support a size-restricted permeability phenotype rather than generalized macromolecular leakage, suggesting that the transient neonatal permeability changes reflect physiological modulation rather than pathological barrier breakdown.

Quantitative spatial analyses further suggested that the vascular and perivascular cell populations labeled by different tracers may represent distinct entities. The perivascular 7HCC⁺ cells observed in neonates showed an average intercellular distance of ~ 45 μm along capillaries and were separated from the endothelium by a narrow gap, indicating that they do not share the vascular basement membrane. Given that the cytoplasmic distance between ECs and PCs in brain capillaries normally measures only 0.1–0.3 μm, including the shared basement membrane [38, 39], the ~ 2.8 μm gap observed here argues against direct localization within the mural cell layer and is more consistent with a perivascular position outside the vascular wall. Their spacing and lack of colocalization with NT500/525-labeled PCs suggest that these 7HCC^+^ cells are unlikely to represent typical PCs. By contrast, FM1-43⁺ cells exhibited no detectable gap from ECs and shared the basement membrane, consistent with a mural/PC-like localization, as further supported by the similar developmental count profiles of FM1-43⁺ and NT500/525-labeled cells. Interestingly, the intercellular spacing of the perivascular macrophages labeled by BSA after mannitol disruption in P25 mice (~ 45 μm) was in a similar range to that of neonatal 7HCC⁺ cells. Although these observations were made at different developmental stages and do not establish cellular identity, they raise the possibility that the transient 7HCC⁺ population reflects a developmentally regulated perivascular cell population. This could include immature fibroblast-like or macrophage-lineage cells that populate the perivascular niche postnatally. Indeed, Col1a1⁺ fibroblast-like cells emerge progressively along larger vessels during the first three postnatal weeks [40], while CNS perivascular macrophages originate from meningeal macrophages and acquire mature markers such as CD206 only after birth [41]. Thus, the transient appearance of 7HCC⁺ perivascular cells in neonates may reflect a dynamic stage in the establishment of the mature perivascular compartment, although marker-based validation will be required to define their identity. Functionally, a transient increase in small-molecule tracer permeability during the neonatal period may serve developmental purposes. The perinatal brain experiences rapid angiogenesis, gliogenesis, and metabolic reprogramming, processes that require dynamic neurovascular exchange. A temporary rise in small-molecule tracer permeability and membrane-associated fluorescence could facilitate redistribution of ions, nutrients, and trophic factors necessary for neuronal growth and metabolic adaptation. In particular, the surge in oxygen and glucose availability after the onset of pulmonary respiration triggers elevated cerebral metabolism and oxidative phosphorylation, increasing local energy demands and potentially altering endothelial homeostasis [33]. Moreover, signaling molecules essential for vascular and neuronal maturation, such as vascular endothelial growth factor, insulin-like growth factor 1, and retinoic acid, peak around birth [32], and transient increases in BBB permeability may enhance their paracrine diffusion between vasculature and neural tissue. Thus, the observed permeability window likely suggests a regulated physiological adaptation that synchronizes vascular, glial, and metabolic maturation rather than an indication of barrier immaturity.

Another key finding of this study is the identification of regional differences in BBB tracer permeability between the striatum and cortex at P25. Functional heterogeneity across brain regions has been suggested by transcriptomic and proteomic profiling of ECs, showing region-specific expression of transporters, junctional proteins, and signaling molecules [24]. Our ISMICAP data provide functional support of this concept at P25: small-molecule permeability was higher in the striatum, whereas membrane-associated FM1-43 fluorescence was lower compared with cortex. Notably, the opposing trends observed for small-molecule tracers and FM1-43 suggest that these probes do not report a single unified measure of BBB permeability, but rather reflect distinct aspects of barrier function. While small-molecule diffusion may be more sensitive to paracellular or junctional properties, FM1-43 dynamics likely capture membrane-associated processes, including endothelial uptake or lipid-membrane interactions. Therefore, the present data do not support a generalized classification of one region as uniformly “more permeable”, but instead point to region-specific differences in tracer-specific barrier behavior at this developmental stage.

These regional differences could be attributed to vascular density, PC coverage, or astrocytic endfeet ensheathment [24]. The apparent striatal specificity of these permeability patterns raises important mechanistic questions. Several, non-mutually exclusive explanations may account for this observation. First, the striatal vasculature may follow a distinct developmental trajectory, with delayed or differential maturation of barrier properties compared to the cortex. Second, regional molecular specialization of endothelial and perivascular cell populations may contribute to these differences, including variation in transporter expression, junctional composition, or astrocytic endfeet coverage. Third, the observed differences may reflect sampling of distinct vascular subpopulations, as capillary architecture and angiogenic states can vary across regions and developmental stages. Region-specific barrier organization could contribute to local variations in metabolite availability and drug penetration, and may underlie the selective vulnerability of certain regions such as the striatum to hypoxic-ischemic and inflammatory insults [16]. Recognizing transient and region-specific BBB permeability windows has clinical relevance. The neonatal period coincides with heightened susceptibility to infection, hypoxia, and inflammation, all of which exacerbate barrier dysfunction [16]. Conversely, such permeability windows may also be therapeutically harnessed to enhance CNS delivery of drugs, antibodies, or gene vectors in early life [16], particularly for managing congenital or perinatal disorders. Exploiting these windows safely, however, will require precise understanding of their timing, magnitude, and molecular regulation to avoid inadvertent toxicity.

While this study provides an integrated functional view of BBB maturation, several limitations should be acknowledged. First, ISMICAP, by design, examines isolated acute brain slices under controlled luminal perfusion conditions and therefore complements, rather than replaces, systemic in vivo permeability assays. This design enables precise control of tracer delivery (concentration and time) and high-resolution visualization of vascular and perivascular tracer behavior, but it does not capture hemodynamic shear stress, systemic clearance, or circulating signals present in vivo. Future studies combining ISMICAP with systemic tracer approaches will be useful to determine how the local permeability dynamics described here relate to whole-animal BBB function.

Second, although the tracer panel was selected to include polar small-molecule tracers and a macromolecular reference that are not established substrates of major BBB efflux transporters, the present experiments were designed to quantify tracer access and extravascular/cellular fluorescence patterns under controlled luminal delivery rather than to mechanistically dissect transporter activity, vesicular trafficking, or endocytic pathways. Therefore, while a dominant transcytotic route is not supported for tracers showing diffuse extravascular fluorescence without detectable endothelial cytoplasmic accumulation, developmental changes in vesicular uptake or tracer-specific cellular accumulation cannot be fully excluded for tracers that show cellular or membrane-associated labeling, such as SR101 and FM1-43.

Third, the longitudinal developmental analysis was focused primarily on the striatum. This region was selected because of its biological relevance to neonatal brain vulnerability and because it allowed reproducible ISMICAP-based measurements across embryonic and postnatal stages. Although we included a cortex-striatum comparison at P25, our findings at this stage indicate that BBB tracer permeability can differ between brain regions. Therefore, the developmental trajectory described here should not be generalized to the entire brain without further validation. Future studies should extend this longitudinal analysis to additional regions, including cortex, hippocampus, and cerebellum, to determine whether the transient neonatal permeability window is region-specific or reflects a broader developmental BBB phenomenon. We note that sex was not controlled across developmental stages, as sex could not be reliably determined at early time points, and only male mice were used at P25 for consistency with and comparability to previously acquired datasets. Therefore, potential sex-dependent differences in BBB permeability cannot be excluded. While temperature is known to influence barrier properties, all experiments were performed under identical conditions. Nevertheless, temperature-dependent effects on absolute BBB permeability cannot be excluded. Finally, the cellular and molecular mechanisms underlying the observed transient neonatal permeability, such as PC maturation, post-translational modification of TJ proteins, efflux transporter regulation, or tracer-specific cellular handling, remain to be elucidated. Addressing these questions will require combining functional imaging with molecular and genetic approaches in follow-up studies.

The functional maturity of the BBB at birth has been debated for decades, largely due to methodological constraints. Many classical approaches such as systemic tracer injections or immunohistochemical detection of endogenous proteins provide integrated, endpoint readouts and often lack the local, controlled delivery needed to dissect permeability dynamics at subcellular resolution [14]. Notably, longitudinal in vivo two-photon imaging through neonatal cranial windows can capture temporal and spatial leakage dynamics in intact cortex [12, 13]; however, ISMICAP complements these approaches by enabling controlled intravascular delivery and direct visualization of tracer-NVU interactions at subcellular resolution under defined conditions. Using this approach, we provide local functional evidence that the embryonic BBB in the striatum is already restrictive but exhibits a transient increase in small-molecule tracer permeability during the early postnatal period.

## Conclusions

Collectively, our findings reveal that BBB maturation is not a linear tightening process but includes a transient neonatal phase of altered, tracer-specific barrier permeability and vascular/perivascular tracer accumulation (Fig. 6). Defining the timing and mechanisms of this phase will be crucial for understanding neonatal brain vulnerability to systemic, hypoxic, or inflammatory insults, and may hold translational potential for targeted CNS drug delivery during early life. Our results provide direct functional measurements supporting temporal and regional differences in BBB permeability during development, while highlighting the need to distinguish tracer-specific barrier permeability from defined junctional, vesicular, or transporter-mediated mechanisms.

## Data Availability

The analyzed datasets in the current study are available from the lead contact upon reasonable request. No codes were generated in this study.

## Materials availability

This study did not generate new unique reagents.

## Code availability

No codes were generated in this study.

## Declarations

### Lead contact

Requests for further information and resources should be directed to and will be fulfilled by the lead contact, Amira Sayed Hanafy (amira.hanafy@uni-bonn.de).

## Abbreviations

7HCC: 7-hydroxycoumarin-3-carboxylic acid
ABC: ATP-binding cassette
BBB: Blood-brain barrier
BSA: Bovine serum albumin
ECs: Endothelial cells
ISMICAP: In situ microperfusion of capillaries
LUT: Look-up tables
mACSF: Modified artificial cerebrospinal fluid
NT500/525: Neurotrace 500/525
NVU: Neurovascular unit
PCs: Pericytes
ROI: Region of interest
sACSF: Standard artificial cerebrospinal fluid
SEM: Standard error of the means
SR101: Sulforhodamine 101
TJs: Tight junctions
TMR: Biocytin-tetramethylrhodamine
